# Cranial autonomic symptoms and response to monoclonal antibodies targeting the Calcitonin gene-related peptide pathway: A real-world study

**DOI:** 10.3389/fneur.2022.973226

**Published:** 2022-09-23

**Authors:** Eleonora De Matteis, Valeria Caponnetto, Alfonsina Casalena, Ilaria Frattale, Amleto Gabriele, Giannapia Affaitati, Maria Adele Giamberardino, Maurizio Maddestra, Stefano Viola, Francesca Pistoia, Simona Sacco, Raffaele Ornello

**Affiliations:** ^1^Department of Biotechnological and Applied Clinical Sciences, University of L'Aquila, L'Aquila, Italy; ^2^Department of Neurology, 'G. Mazzini' Hospital, Teramo, Italy; ^3^Department of Child Neurology and Psychiatry, University of Rome “Tor Vergata”, Rome, Italy; ^4^Neurology Service, 'SS. Annunziata' Hospital, Sulmona, Italy; ^5^Department of Medicine and Science of Aging, 'G. D'Annunzio' University, Chieti, Italy; ^6^Department of Neurology, 'Floraspe Renzetti' Hospital, Lanciano, Italy; ^7^Department of Neurology, 'San Pio da Pietralcina' Hospital, Vasto, Italy

**Keywords:** migraine, anti-CGRP monoclonal antibodies, trigeminovascular system, CAS, cranial autonomic symptoms

## Abstract

**Objective:**

Cranial autonomic symptoms (CAS), including conjunctival injection, tearing, nasal congestion or rhinorrhea, eyelid edema, miosis or ptosis, and forehead or facial sweating ipsilateral to headache, are often reported by patients with migraine during headache attacks. CAS is a consequence of the activation of the trigeminovascular system, which is the target of monoclonal antibodies acting on the CGRP pathway. Therefore, we hypothesized that patients with CAS might have higher trigeminovascular activation than those without CAS leading to a better response to anti-CGRP treatments.

**Methods:**

We performed a prospective analysis including patients with episodic or chronic migraine treated with anti-CGRP monoclonal antibodies (i.e., erenumab, fremanezumab, and galcanezumab) between 2019 and 2021. The observation period included a 12-week baseline before treatment with anti-CGRP antibodies and a 12-week treatment follow-up. We evaluated the prevalence of CAS in our cohort and compared disease characteristics and treatment response (i.e., 12-week monthly headache days and 0–29, 30–49, 50–74, 75–99, and 100% monthly headache days reduction from baseline) among patients with and without CAS using the χ^2^ test, Kruskal–Wallis test, and Mann–Whitney U-test.

**Results:**

Out of 136 patients, 88 (65%) had CAS. Both patients with and without CAS reported a significant decrease in monthly headache days from baseline. During the 12-week follow-up, the median difference in monthly headache days from baseline was higher in patients with CAS (-10, IQR−15 to−6) than in those without CAS (6, IQR 12 to 3; *P* = 0.009). However, the proportions of patients with 0 to 29, 30 to 49, 50 to 74, 75 to 99, and 100% response rates did not differ between the two groups.

**Conclusions:**

In our cohort, the presence of CAS was associated with a greater response to monoclonal antibodies targeting the CGRP pathway. CAS could be a clinical marker of trigeminovascular activation and thus be related to a better response to CGRP treatments.

## Introduction

Migraine is the second cause of disability worldwide mainly affecting young and middle-aged women (1). The International Classification of Headache Disorders (ICHD-3) defines migraine as a unilateral, mainly pulsating headache worsened by physical activity and often associated with nausea and/or phonophobia (2). Cranial autonomic symptoms (CAS) include conjunctival injection or lacrimation, nasal congestion and/or rhinorrhea, eyelid edema, forehead and facial sweating, and miosis and/or ptosis; while they are mandatory for the diagnosis of trigeminal autonomic cephalalgias and appear unilateral to the side of headache, they are not required for the diagnosis of migraine according to the ICHD-3 (2) but have been described in up to 50% of the cases with a mostly bilateral appearance. CAS occur due to the activation of the trigeminal autonomic reflex, a physiological response to nociceptive stimuli determining a parasympathetic reaction (3): a stimulus on the face, neck, or eye is transmitted through trigeminal afferents from the periphery to the trigeminal cervical complex in the brainstem, and then up to thalamus and cortical areas. The trigeminal cervical complex leads to the activation of parasympathetic fibers of the superior salivatory nucleus in the reticular formation, near the motor facial nucleus, which is the efferent arc responsible for CAS through the activation of the sphenopalatine ganglion and greater superficial petrosal nerve (3–5).

The trigeminal cervical complex is a relay, where signals run from central to peripheral structures of the nervous system and the other way round. Migraine initiates in the Central nervous system (CNS)—dorsal pons, hypothalamus, and thalamus—from where pain signals reach the trigeminal ganglion activating the trigeminovascular system, then the trigeminal cervical complex and back to the initial CNS areas (6, 7). The trigeminal ganglion in turn releases Calcitonin gene-related peptide (CGRP), a peptide playing a key role in the genesis of migraine pain (8) and likely involved in CAS. Indeed, a study demonstrated that patients with migraine and CAS have higher CGRP levels in the external jugular blood during an attack compared with patients without CAS (9). Given the indirect evidence that CAS could be related to the trigeminal autonomic reflex led by CGRP release from the trigeminal ganglion, we hypothesized that the presence of CAS could be associated with a greater response to treatments acting on the CGRP. So far, no real-world study evaluated the effectiveness of monoclonal antibodies (mAbs) targeting the CGRP pathway according to the presence of CAS (10). Hence, we investigated a possible association between CAS and mAbs response in a real-world setting.

## Methods

### Study design, participants, and setting

This prospective open-label study followed the Strengthening the Reporting of Observational Studies in Epidemiology (STROBE) guidelines (11) and the protocol of a real-world study with published data (12). We included patients aged 18 years or more consecutively treated with mAbs acting on the CGRP pathway in multiple Headache Centers of the Abruzzo region in Central Italy (Avezzano, L'Aquila, Sulmona, Teramo, Chieti, Lanciano, and Vasto) from January 2019 to August 2021. The Internal Review Board of the University of L'Aquila cleared the study with approval number 44/2019. All patients signed informed consent.

Until July 2020, manufacturers provided mAbs upon request from the Headache Centers to treat patients with migraine with or without aura according to the ICHD-3 criteria (2), ≥4 monthly headache days (MHDs), and ≥ 2 prior treatment failures, as suggested by the European Headache Federation and the American Headache Society guidelines (13, 14). After July 2020, physicians prescribed the treatment according to national reimbursement criteria [i.e., diagnosis of episodic or chronic migraine according to ICHD-3 criteria (2), at least eight disabling headache days for the last three consecutive months, MIDAS score ≥11, 6-week treatment failure with or contraindication to three classes of oral preventative among beta-blockers, onabotulinumtoxinA, tricyclic antidepressants, and antiepileptic drugs] (15). During the treatment period with mAbs, patients were allowed to start, continue, or discontinue concurrent oral migraine preventives. Patients with medication overuse did not undergo detoxification before treatment with mAbs according to current recommendations and clinical experience (13).

### Study procedures, data collection, and variables

The presence of CAS was prospectively assessed at the beginning of treatment with mAbs through clinical interview; for the classification of CAS (conjunctival injection, tearing, nasal congestion or rhinorrhea, eyelid edema, miosis or ptosis, forehead or facial sweating ipsilateral to headache), we referred to a previous prospective study (16). We considered patients with CAS those referring to at least one CAS in at least one headache attack. The efficacy of mAbs was assessed by using prospective headache diaries.

The study comprised a baseline period (i.e., the 12 weeks preceding treatment with mAbs) and a 12-week follow-up (i.e., 12 weeks of treatment with mAbs targeting the CGRP pathway). We recorded patients' age, sex, migraine type (episodic or chronic), medication overuse, presence of aura, and disease duration at baseline. Patients reported monthly headache days (MHDs) on specific diaries throughout baseline and follow-up. During baseline and follow-up clinical interviews, patients reported the frequency of CAS on a five-level Likert scale (i.e., never, rarely, sometimes, often, always as CAS occurring in none, 25, 50, 75, and 100% of the attacks, respectively). We computed median MHDs as a 4-week mean of the 12-week baseline and the 12-week follow-up. Similarly, we computed the 12-week response rate as 0 to 29, 30 to 49, 50 to 74, 75 to 99, and 100% median MHDs reduction throughout the follow-up period from baseline. Treatment was recommended to patients for at least 12 weeks; however, before the issue of reimbursement criteria, patients were allowed to stop the treatment before week 12 due to perceived ineffectiveness. Patients reporting severe adverse events before week 12 of also stopped the treatment. Patients stopping the treatment before week 12 were considered in the analyses and 12-week MHDs were estimated from the last available value, following a “last observation carried forward” approach. A “headache day” was defined as a day when patients reported any headache, migraine, or non-migraine in their diaries. Data were entered in an electronic anonymized database created on the Research Electronic Data Capture (REDCap) software for the analyses hosted at the University of L'Aquila (17, 18).

### Statistical analyses

We reported descriptive statistics about patients' demographics and disease characteristics (MHDs, presence, and type of CAS). Categorical data were reported as numbers and percentage, while continuous data were reported as the median and interquartile range (IQR). We did not perform a formal sample size calculation as we were based upon a convenience sample; therefore, we used conservative non-parametric tests: the χ^2^ test to compare categorical data, and the Mann–Whitney U-test or Kruskal–Wallis test to compare continuous data.

## Results

We included 136 patients treated with mAbs for 12 weeks in our Headache centers: 116 (85.3%) were female, the median age was 49 (IQR 41–56.7), median disease duration was 26.5 years (IQR 17–38.5), 105 (77.2) suffered from chronic migraine, 80 (58.8%) had medication overuse headache as comorbidity, and 24 (17.6%) reported aura. Baseline characteristics were similar between patients with and without CAS (Table 1). One-hundred and sixteen patients (86%) received erenumab, 10 (7.3 %) fremanezumab, and nine (6.7%) galcanezumab. Two patients (1.4%) stopped treatment before week 12 due to perceived ineffectiveness but were still included in the present analysis; none stopped the treatment due to severe adverse events.

**Table 1:** Characteristics of the overall study population and the subgroups: patients with and without cranial autonomic symptoms.

**Baseline characteristics**	**Overall study population (*N* = 136)**	**Patients with CAS (*N* = 88)**	**Patients without CAS (*N* = 48)**	***P*-value**
Age, median (IQR)	49 (41–56.7)	49 (42.2–56)	49 (38.5–57.7)	0.922
Disease duration, median (IQR)	26.5 (17–38.5)	26 (17–40)	28 (18–35)	0.762
Female, *N* (%)	116 (85.3)	77 (87.5)	39 (81.3)	0.325
Chronic migraine, *N* (%)	105 (77.2)	71 (80.7)	34 (70.8)	0.149
Medication overuse, *N* (%)	80 (58.8)	55 (62.5)	25 (52.1)	0.208
MHDs, median (IQR)	18 (12–27.2)	18 (12.5–28)	15 (12–20.7)	0.108
Aura, *N* (%)	24 (17.6)	18 (20.5)	6 (12.5)	0.234

IQR, interquartile range; MHDs, monthly headache days; N, number; CAS, cranial autonomic symptoms.

**Figure 1:**
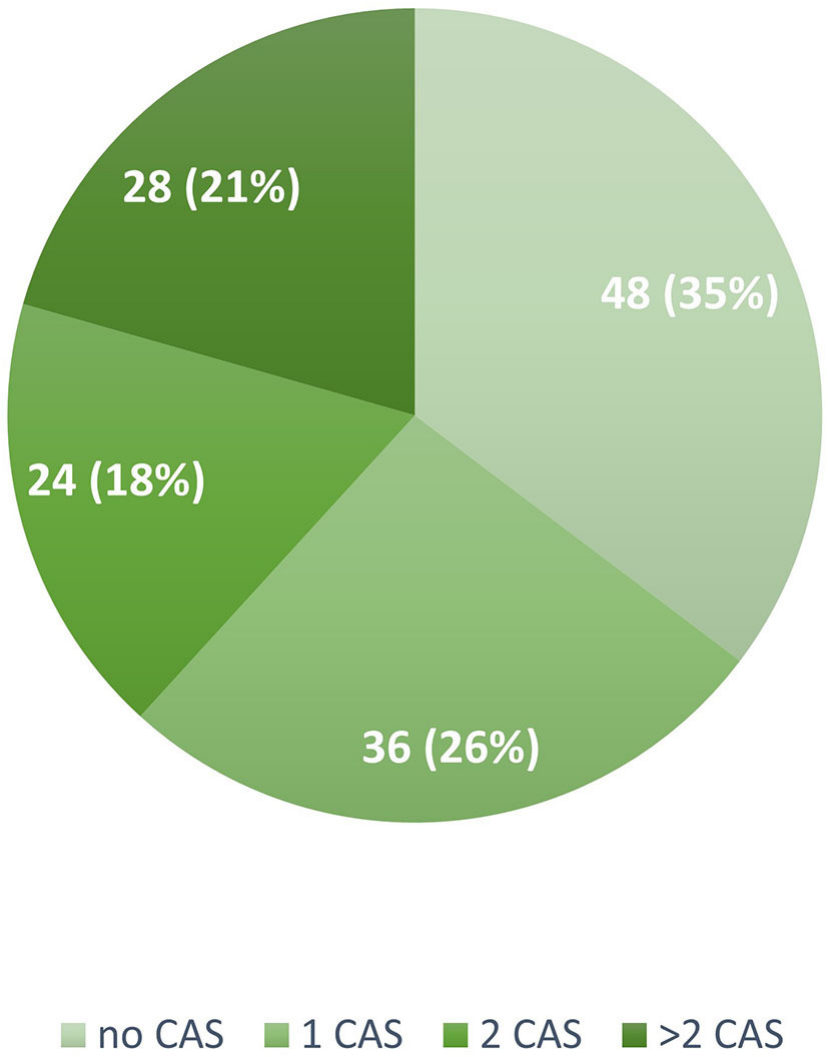
Number and proportion of patients reporting none, one, two, or more cranial autonomic symptoms in the study population (*N* = 136). CAS, Cranial Autonomic Symptoms.

**Figure 2:**
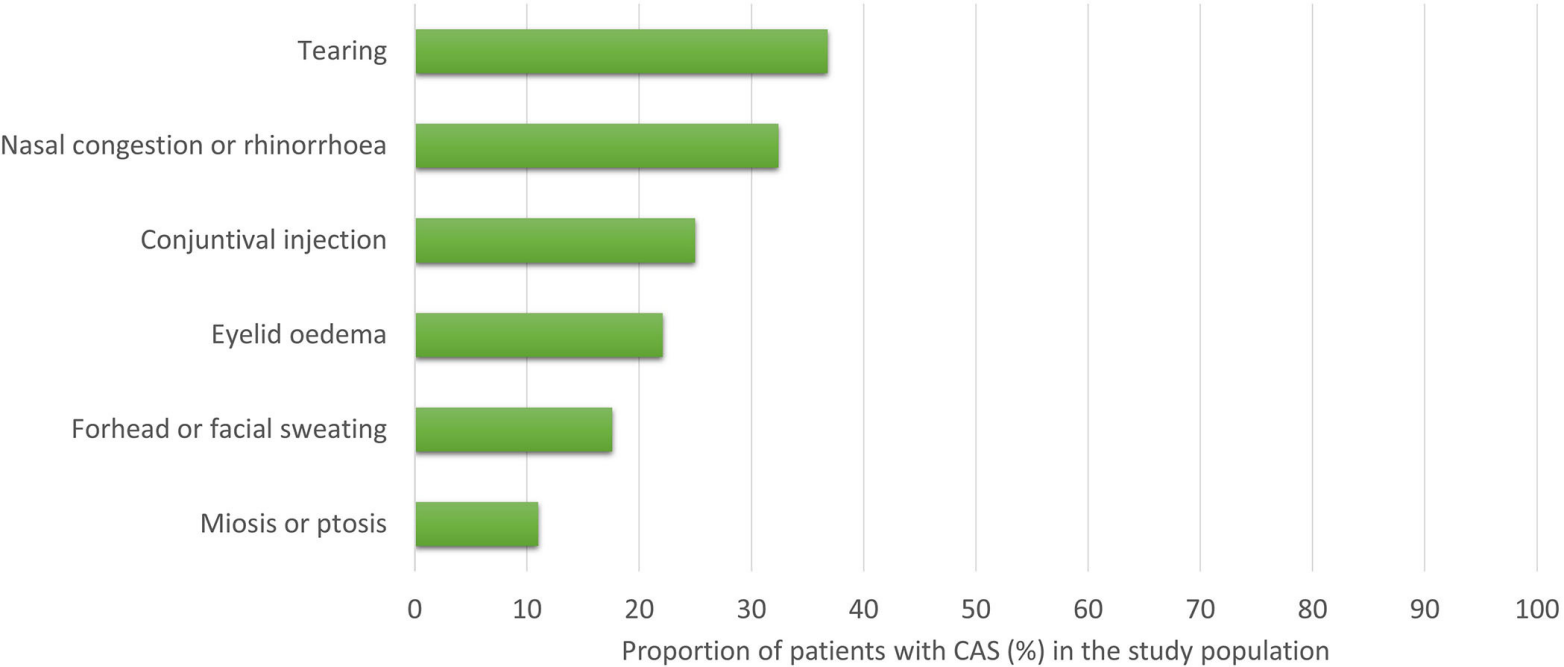
Frequency of cranial autonomic symptoms (*N* = 88). CAS, Cranial Autonomic Symptoms.

In our cohort, 88 patients (64.7%) reported at least one CAS: 36 (41%) reported one CAS, 24 (27%) two CAS, and 28 (32%) more than two CAS (Figure 1). The frequency of the different types of CAS is detailed in Figure 2. A total of 66 of the 91 (67%) patients receiving mAbs before the issue of national reimbursement criteria in July 2020 reported at least one CAS compared with 22 of the 45 (48%) patients who received the treatment after that date (*P* = 0.007): disease characteristics were similar across the two groups (Supplementary Table 1).

**Figure 3:**
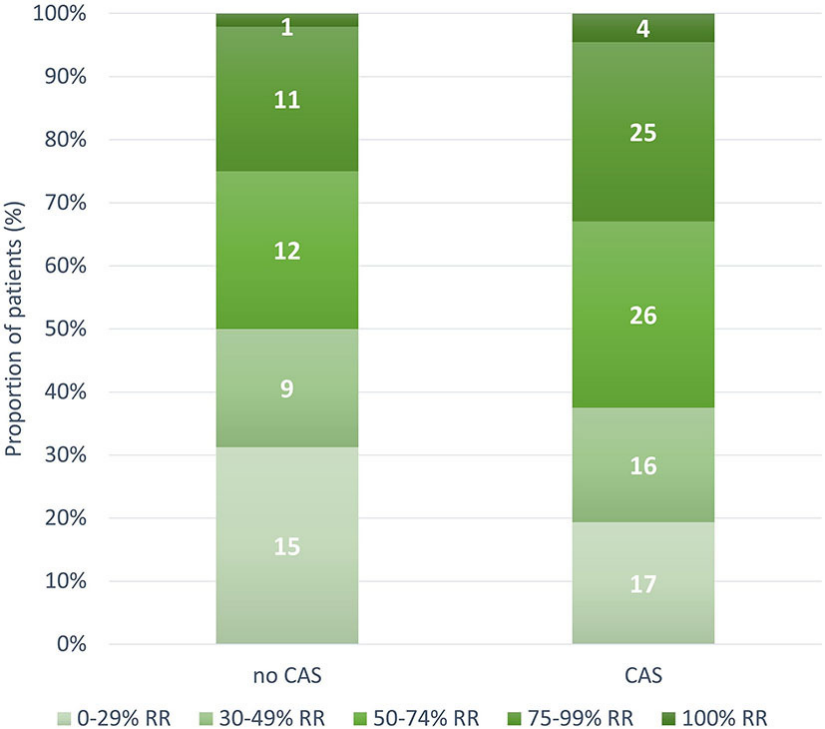
Number and proportion of patients with 0 to 29, 30 to 49, 50 to 74, 75 to 99, and 100% response rate according to the presence of cranial autonomic symptoms (*P* = 0.561). CAS, Cranial Autonomic Symptoms; RR, response rate.

**Figure 4:**
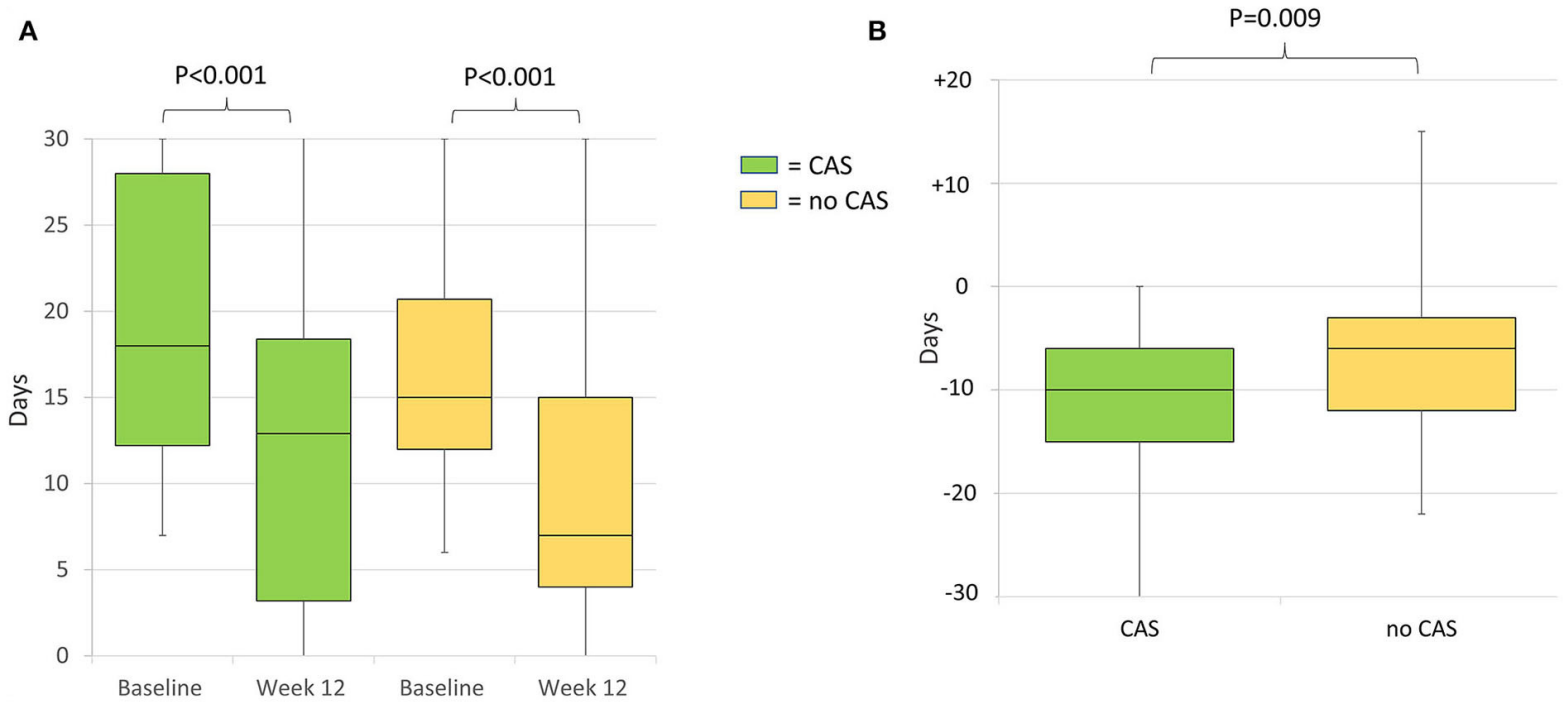
**(A)** Median monthly headache days at baseline and week 12 of treatment of patients with and without cranial autonomic symptoms. Baseline monthly headache days did not differ between the groups (*P* = 0.108). **(B)** Median difference in monthly headache days from baseline to week 12 in patients with and without cranial autonomic symptoms. CAS, Cranial Autonomic Symptoms.

During the 12-week follow-up, 0 to 29, 30 to 49, 50 to 74, 75 to 99, and 100% response rate was achieved by 32 (23.5%), 25 (18.4%), 38 (27.9%), 36 (26.5%), and five (3.7%) patients, respectively. The 30 to 49, 50 to 74, 75 to 99, and 100% response rates were numerically higher in patients with CAS than in those without; however, the between-group difference was not significant (Figure 3). MHDs decreased from a median of 18 (IQR 12–27.2) to 7 (IQR 4–12.7; *P* < 0.001); median difference in MHDs from baseline to 12 weeks was higher in patients with CAS than those without, −10 (IQR −15 to −6) and −6 (IQR −12 to −3), respectively (*P* = 0.009) (Figures 4A,B).

Out of 88 patients with CAS, 80 patients (91%) reported the frequency of symptoms throughout the entire study period. MAbs response did not differ according to the frequency and the number of CAS (Supplementary Results; Supplementary Tables 2, 3; Supplementary Figures 1–4).

## Discussion

In our cohort, two-thirds of patients suffering from different forms of migraine (episodic, chronic, with or without aura) reported at least one CAS. The high frequency of CAS was expected because a similar rate was reported in other studies, where those symptoms had been reported by up to 74% of patients with migraine (5, 19). A high CAS frequency might be due to the high number of patients with severe forms of disease and treatment refractoriness followed in tertiary headache Centers such as the sites of this and the aforementioned studies. At variance with previous evidence showing that CAS were associated with more severe disease characteristics, such as headache frequency and intensity (5, 20, 21), the frequency of MHDs at baseline did not differ between those with and without CAS. Again, this may be due to the highly selected cohort.

MAbs targeting the CGRP pathway significantly reduced MHDs and more remarkably in patients with CAS. The higher efficacy of treatment in these patients might be due to greater activation of the trigeminovascular system, where the CGRP plays a key role in mechanisms underlying migraine pain—a mediator of nociception, neuroinflammation, and vasodilation (6). The presence of CAS even in other headache disorders and in human experimental trigeminal pain models supports the hypothesis of greater activation of the trigeminovascular system in patients reporting those symptoms. CAS might be an epiphenomenon of trigeminal pain and a possible marker for trigeminal pain processing in general. The blockade of CGRP-related pathways through mAbs, gepants, and ditans or other treatments with a supposed action at this level, such as onabotulinumoxinA (22) and triptans (23), might inhibit the trigeminal-autonomic reflex, which consists of functional connections between trigeminal afferences and parasympathetic efferences.

Several studies have demonstrated how CAS respond to triptans, such as sumatriptan and rizatriptan, and how patients with these symptoms report better responses to triptans (20). Patients with CAS, compared with those without, showed a greater response to rizatriptan which inhibits the CGRP release from trigeminal presynaptic terminals; responders to triptans also had increased blood levels of CGRP, NKA, and VIP during headache (9). These peptides were higher even in responders to onabotulinumtoxin A than in non-responders (24). We speculate that patients with a strong trigeminovascular system activation more frequently report CAS and better respond to treatments acting on the CGRP pathway, also considering recent data that showed an association among CAS, allodynia, and osmophobia with (25) triptans (26) and erenumab responses. However, the efficacy of mAbs targeting the CGRP pathway in patients with cluster headaches, where the presence of CAS is pathognomonic, is controversial. Some case series proved a disease remission both in episodic and chronic cluster headaches (27, 28); whereas most RCTs (NCT02964338, NCT02438826, NCT02945046, NCT03107052) failed to show the superiority of treatment to placebo (29), and NCT02397473 was the sole showing a significantly lower number of attacks in patients treated with galcanezumab 300 mg monthly rather than placebo (30). Treatment regimens and patient selection might explain different results across the studies. Moreover, CAS might be epiphenomena of slightly different and partly unknown mechanisms in the migraine and cluster headaches.

The advent of highly effective and migraine-specific preventatives such as mAbs imposes a new tailored-made approach to migraine therapy, which should consider patients' responses and needs. Clinical predictors of response to preventatives and acute treatments targeting the CGRP pathway could help to maximize the cost-effectiveness of these drugs. Real-world studies proved that some peculiarities of the attacks predict mAbs response such as unilateral pain, baseline MHDs, acute medication intake, number of prior treatment failures, allodynia, and psychiatric comorbidities (12, 25, 31–33). Further studies showed that CAS predict response to triptans (20, 34, 35). We were not able to define CAS as predictors of response to anti-CGRP pathway mAbs because of the treatment's great efficacy even in those without CAS, but we showed that these symptoms were solely associated with a higher MHDs decrease among baseline disease characteristics. Our data require cautious interpretation as we did not evaluate CGRP serum levels in different groups of patients—responders/non-responders and patients with and without CAS. Therefore, we could not define CAS as a clinical criterion to guide treatment choice, but future studies might support their use to predict treatment response.

To our knowledge, this is the first study evaluating the efficacy of mAbs according to the presence of CAS. We support the idea that the CGRP pathway plays a role in the genesis of these symptoms, which might be useful in phenotyping migraine attacks. Changes in prescription criteria for mAbs affected the presence of CAS but not the other baseline disease characteristics, thus, they did not influence patient selection. We prescribed mAbs to those suffering from the most severe forms of migraine before and after the issue of national reimbursement criteria. The small sample size might have affected the results. Also, we did not distinguish between monthly headache days and migraine days, thus we did not precisely assess changes in CAS frequency according to migraine attacks; the collection of CAS frequency through patients' interviews might have been prone to a recall bias. We did not collect data such as number of triptans or acute medications, concomitant preventive treatments, intensity of the attacks, and localization of pain and CAS (i.e., unilateral or bilateral CAS); the short follow-up and the 12-week unique assessment timepoint did not allow to identify early and late responders to the treatment and CAS frequency according to this response. Larger studies might confirm our results and show a possible statistical difference in response rate according to the presence of CAS.

In conclusion, our data suggest the clinical relevance of investigating CAS in patients with migraine with and without aura as they might signal a high trigeminal activation and thus be associated with a high response to treatments targeting the CGRP. Our hypothesis stemmed from real-world data that needs confirmation from basic science.

## Data availability statement

The raw data supporting the conclusions of this article will be made available by the authors, without undue reservation.

## Ethics statement

The studies involving human participants were reviewed and approved by Internal Review Board (IRB), University of L'Aquila, L'Aquila. The patients/participants provided their written informed consent to participate in this study.

## Author contributions

RO, SS, and ED conceived the study and its design, performed the acquisition, analysis, interpretation of data, and drafted the manuscript. AC, IF, VC, AG, GA, MG, MM, SV, and FP collected data and substantively revised the manuscript. All authors approved the final manuscript.

## Funding

This work was supported by the Department of Biotechnological and Applied Clinical Sciences, University of L'Aquila, L'Aquila, Italy.

## Conflict of interest

Author RO has received sponsorship to attend meetings from Novartis and Teva. Author SS had a financial relationship (lecturer or member of advisory board) with Abbott, Allergan, Novartis, Teva, and Eli Lilly. Author GA has received funds for congress participation from Innovet Italia Srl, Epitech Group, and Lusofarmaco. Author MG received funds for congress participation from IBSA. The remaining authors declare that the research was conducted in the absence of any commercial or financial relationships that could be construed as a potential conflict of interest.

## Publisher's note

All claims expressed in this article are solely those of the authors and do not necessarily represent those of their affiliated organizations, or those of the publisher, the editors and the reviewers. Any product that may be evaluated in this article, or claim that may be made by its manufacturer, is not guaranteed or endorsed by the publisher.
