# Supplementary material for: Cranial autonomic symptoms and response to monoclonal antibodies targeting the Calcitonin gene-related peptide pathway: A real-world study
Source: Front Neurol. 2022 Sep 23;13:973226. doi: 10.3389/fneur.2022.973226 (PMC9538972; doi:10.3389/fneur.2022.973226)
Supplement: Supplementary file 1 [file Data_Sheet_1.docx]

# SUPPLEMENTARY RESULTS

Among the 80 (91%) patients reporting the frequency of CAS during migraine attacks, symptoms occurred ‘rarely’ in 19 (23.8%), ‘sometimes’ in 23 (28.8%), ‘often’ in 21 (26.3%), and ‘always’ in 17 (21.3%). Median baseline and 12-week follow-up MHDs were similar across the groups (P=0.385 and P=0.210 respectively) (Supplementary Table 2). Similarly, there was no statistically significant difference across the groups in terms of median MHDs difference from baseline (Supplementary Figure 1) and 29%, 30-49%, 50-74%, 75%-99% and 100% response rate (Supplementary Figure 2).

According to the number of CAS, we divided patients in 3 groups: those with none, 1, 2 or ≥ 2 CAS. The groups did not differ in terms of median baseline (P=0.250) and 12-week follow-up MHDs (P=0.840) (Supplementary Table 3), median MHDs difference from baseline (Supplementary Figure 3) and 0-29%, 30-49%, 50-74%, 75%-99%, and 100% response rates (Supplementary Figure 4).

# SUPPLEMENTARY TABLES

**Supplementary Table 1:** characteristics of patients treated with monoclonal antibodies targeting the CGRP pathway before and after the issue of national reimbursement criteria in July 2020

| **Characteristics** | **Patients receiving mAbs before July 2020 (N=91)** | **Patients receiving mAbs from July 2020 (N=45)** | **P value** |
| --- | --- | --- | --- |
| Age, median (IQR) | 50 (44-59) | 46 (35-55) | **0.025** |
| Disease duration, median (IQR) | 28 (17-40) | 25.5 (18.5-37.7) | 0.781 |
| Female, N (%) | 79 (87%) | 37 (82%) | 0.477 |
| Chronic migraine, N (%) | 71 (78%) | 34 (75%) | 0.922 |
| Medication overuse, N (%) | 54 (60%) | 26 (58%) | 0.978 |
| Aura, N (%) | 20 (21%) | 4 (8%) | 0.066 |
| MHDs, median (IQR) | 18 (12-28) | 15 (12-23) | 0.181 |

*CAS*- cranial autonomic symptoms; *IQR*- interquartile range; *mAbs*-monoclonal antibodies; *MHDs*-monthly headache days; *N-*number.

**Supplementary Table 2:** median monthly headache days at baseline and 12-week follow-up according to frequency of cranial autonomic symptoms.

| Frequency of CAS (N=80) | Baseline MHDs, median (IQR) | 12-week MHDs  median (IQR) |
| --- | --- | --- |
| Rarely (N=19) | 20 (16-28) | 12 (3-15) |
| Sometimes (N=23) | 18 (11-25) | 6 (4-10) |
| Often (N=21) | 18 (10-25) | 6 (3-9) |
| Always (N=17) | 25 (14-30) | 10 (4.5-15) |

*CAS*- cranial autonomic symptoms; *IQR*- interquartile range; *MHDs*-monthly headache days; *N-*number.

**Supplementary Table 3:** median monthly headache days at baseline and 12-week follow-up according to the number of cranial autonomic symptoms.

| **Number of CAS** | **Baseline MHDs, median (IQR)** | **12-week MHDs**  **median (IQR)** |
| --- | --- | --- |
| None (N=48) | 15 (12-20.7) | 7 (4-15) |
| 1 CAS (N=36) | 20 (12.2-28.7) | 6 (3-13.7) |
| 2 CAS (N=24) | 20 (15.2-10) | 7.5 (4-11.7) |
| ≥ 2 CAS (N=28) | 18 (12-27.5) | 6.5 (3-10) |

*CAS*-cranial autonomic symptoms*; IQR*- interquartile range; *MHDs*-monthly headache days; *N-*number

# SUPPLEMENTARY FIGURES

**Supplementary Figure 1:** Median difference in monthly headache days from baseline to week 12 in patients with cranial autonomic symptoms (N=80) according to the frequency of the symptoms P=0.210.


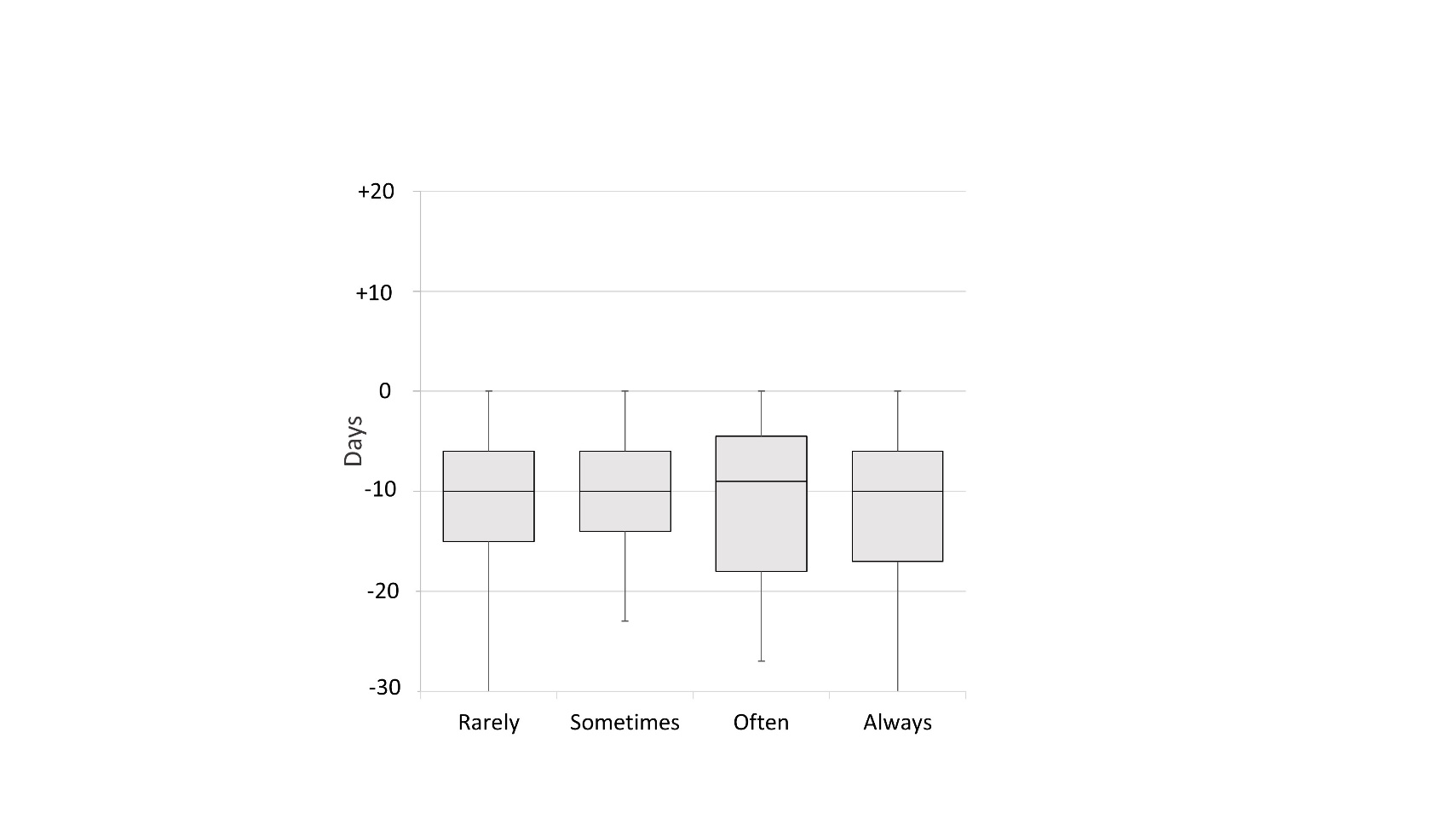


**Supplementary Figure 2:** Number and proportion of patients with 0-29%, 30-49%, 50-74%, 75%-99% and 100% response rate according to frequency of cranial autonomic symptoms (P=0.852).


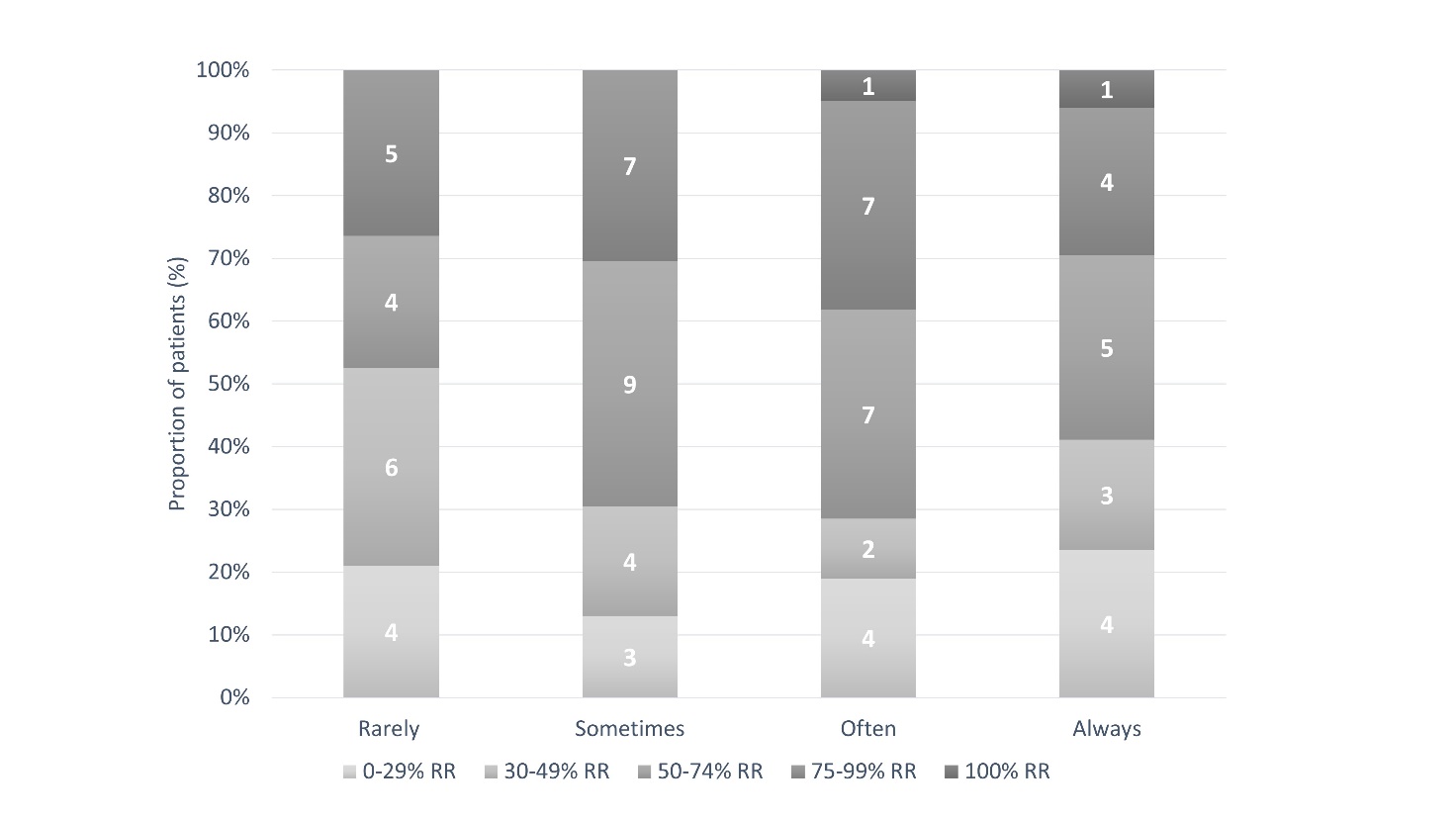


Abbreviations: RR-response rate.

**Supplementary Figure 3:** Median difference in monthly headache days from baseline to week 12 according to the number of cranial autonomic symptoms (P=0.845).

**
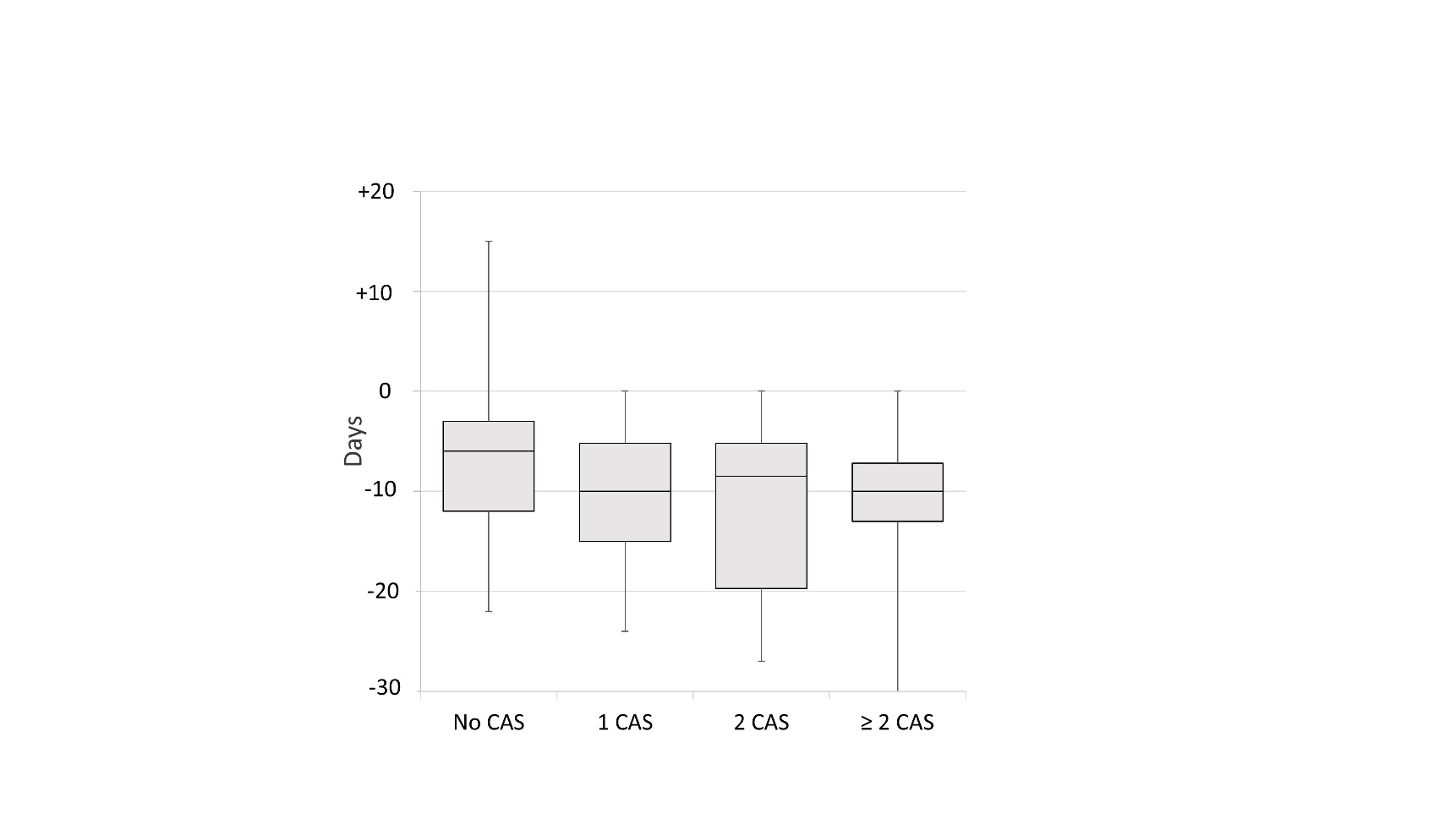
**

Abbreviations: CAS-cranial autonomic symptoms.

**Supplementary Figure 4:** Number and proportion of patients with 0-29%, 30-49%, 50-74%, 75%-99%, and 100% response rate according to the number of cranial autonomic symptoms (P=0.883).


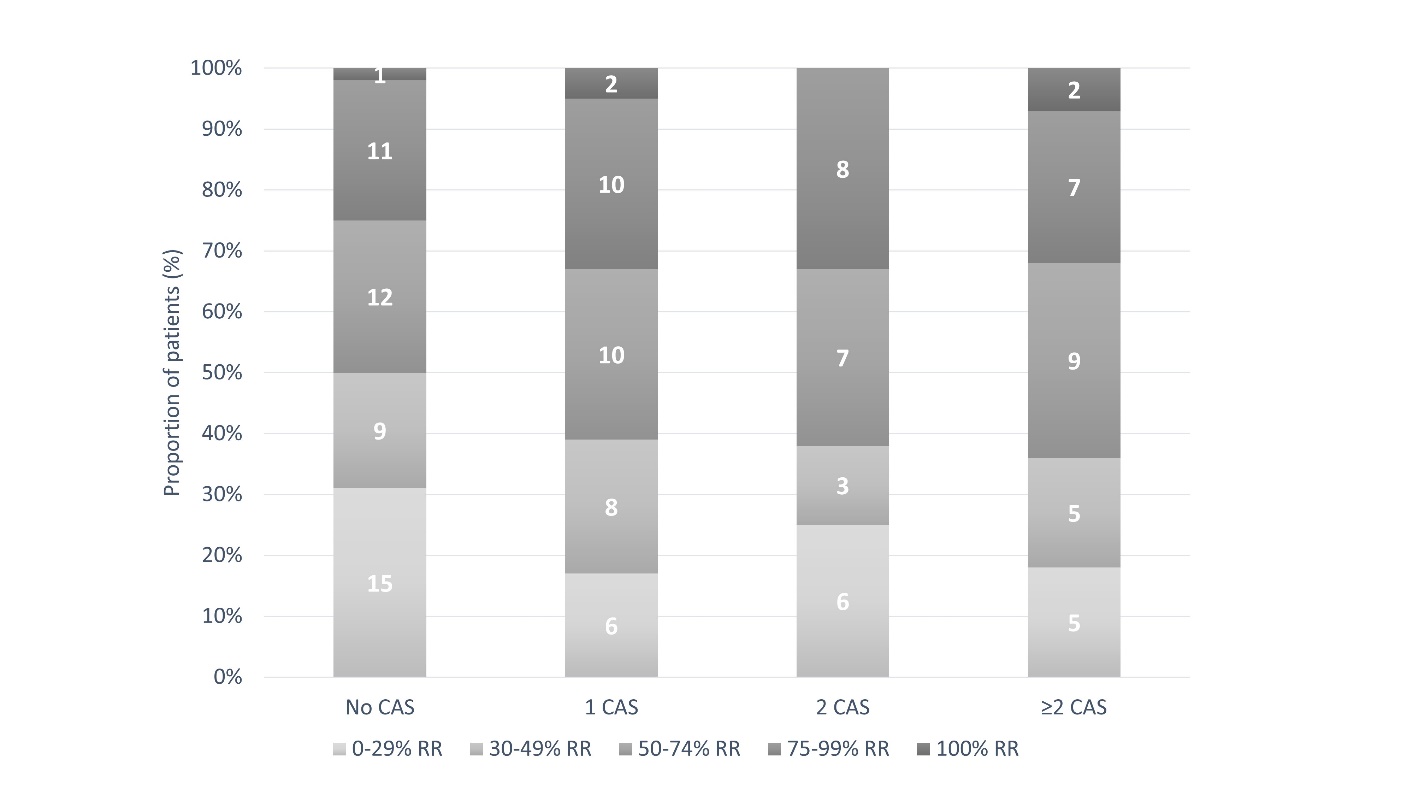


Abbreviations: CAS-cranial autonomic symptoms, RR-response rate.
